# Tumor BRCA Test for Patients with Epithelial Ovarian Cancer: The Role of Molecular Pathology in the Era of PARP Inhibitor Therapy

**DOI:** 10.3390/cancers11111641

**Published:** 2019-10-24

**Authors:** Caterina Fumagalli, Federica Tomao, Ilaria Betella, Alessandra Rappa, Mariarosaria Calvello, Bernardo Bonanni, Loris Bernard, Fedro Peccatori, Nicoletta Colombo, Giuseppe Viale, Massimo Barberis, Elena Guerini-Rocco

**Affiliations:** 1 Division of Pathology, European Institute of Oncology (IEO), Istituto di Ricovero e Cura a Carattere Scientifico (IRCCS), 20141 Milan, Italy; caterina.fumagalli@ieo.it (C.F.); alessandra.rappa@ieo.it (A.R.); giuseppe.viale@ieo.it (G.V.); elena.guerinirocco@ieo.it (E.G.-R.); 2 Department of Obstetric, Gynecologic and Urologic Sciences, University of Rome “Sapienza”, 00161 Rome, Italy; federica.tomao@uniroma1.it; 3 Division of Gynecologic Surgery, IEO, European Institute of Oncology IRCCS, 20141 Milan, Italy; ilaria.betella@ieo.it; 4 Division of Cancer Prevention and Genetics, IEO, European Institute of Oncology IRCCS, 20141 Milan, Italy; mariarosaria.calvello@ieo.it (M.C.); bernardo.bonanni@ieo.it (B.B.); 5 Clinical Genomics Lab, IEO, European Institute of Oncology IRCCS, 20141 Milan, Italy; loris.bernard@ieo.it; 6 Division of Gynecologic Oncology, IEO, European Institute of Oncology IRCCS, 20141 Milan, Italy; fedro.peccatori@ieo.it (F.P.); nicoletta.colombo@ieo.it (N.C.); 7 School of Medicine and Surgery, Università degli Studi di Milano-Bicocca, 20126 Milan, Italy; 8 Department of Oncology and Hemato-Oncology, University of Milan, 20141 Milan, Italy

**Keywords:** *BRCA1/2*, ovarian cancer, next-generation sequencing

## Abstract

The PARP inhibitor olaparib has been approved in the maintenance setting of platinum-sensitive epithelial ovarian cancer patients with germline or somatic *BRCA1/2* mutation. Therefore, the availability of a tumor BRCA test has become a clinical need. We report the results of the clinical implementation of a tumor BRCA test within the frame of an institutional workflow for the management of patients with nonmucinous and nonborderline epithelial ovarian cancer. In total, 223 patients with epithelial ovarian cancer were prospectively analyzed. *BRCA1/2* status was evaluated on formalin-fixed, paraffin-embedded tumor specimens using next-generation sequencing technology. The tumor BRCA test had a success rate of 99.1% (221 of 223 successfully analyzed cases) and a median turnaround time of 17 calendar days. Among the 221 cases, *BRCA1* or *BRCA2* pathogenic/likely pathogenic mutations were found in 62 (28.1%) cases and variants of uncertain significance in 25 (11.3%) cases. The concordance rate between tumor BRCA test results and germline *BRCA1/2* status was 87%, with five cases harboring pathogenic/likely pathogenic somatic-only mutations. The next-generation, sequencing-based tumor BRCA test showed a high success rate and a turnaround time compatible with clinical purposes. The tumor BRCA test could be implemented in a molecular diagnostic setting and it may guide the clinical management of patients with epithelial ovarian cancer.

## 1. Introduction

Epithelial ovarian cancer (EOC) is the seventh most common cancer among women worldwide [1], frequently diagnosed in an advanced stage and with an aggressive clinical course [2]. Despite surgical resection and standard chemotherapy, the frequency of disease relapse at 5 years may reach 40–75% [3]. The introduction of poly (adenosine diphosphate (ADP)-ribose) polymerase inhibitors (PARPi) has marked a crucial step forward in the treatment of these patients. Up until now, three PARPi—olaparib, niraparib, rucaparib—have received the Food and Drug Administrations (FDA) and European Medicines Agency (EMA) approvals in different clinical settings [4,5,6]. Olaparib has been approved in Europe for the maintenance of platinum-sensitive recurrent EOCs patients with a somatic or germline *BRCA1/2* mutation [4]. Moreover, based on the recent striking results of SOLO 1 trial, olaparib will be used as maintenance therapy even for women with newly diagnosed advanced EOC harboring *BRCA1/2* mutations [7]. Therefore, the evaluation of *BRCA1* and *BRCA2* status is nowadays mandatory for all patients with advanced nonmucinous EOC to guide the therapy decision. In this setting, the analysis of *BRCA1/2* should meet precise requirements [8,9]. First, the test should be performed on tumor specimens, enabling to capture both somatic and germline alterations; second, the test should be suitable for formalin-fixed, paraffin-embedded (FFPE) specimens, available in all the pathology laboratories; third, the test turnaround time should be consistent with treatment decision purposes; finally, the test should be included in a comprehensive and multidisciplinary clinical management of women with EOC.

In the last years, different studies have reported on the implementation and validation of next-generation sequencing (NGS) platforms to assess the *BRCA1/2* status in FFPE specimens [10,11,12]. Although these NGS panels allowed a shorter test turnaround time (TAT) as compared with standard Sanger sequencing, the use of these assays in a clinical diagnostic scenario may be challenging [8,9].

In this study, we prospectively analyzed the tumor *BRCA1/2* status in FFPE samples from 223 patients with EOC, aiming (i) to ascertain the feasibility of the tumor NGS-based BRCA test in the clinical setting, and (ii) to evaluate the prevalence of tumor *BRCA1* and *BRCA2* mutations in a single-institution cohort of women with epithelial ovarian cancers.

## 2. Results

### 2.1. Tumor BRCA Test Performance

All the FFPE tumor samples were considered adequate for the analysis according to the tumor cell content (more than 10%) and the DNA yield (more than 10 ng). In two cases (0.9%), the NGS analysis failed due to low NGS run quality parameters and FFPE artifacts. Overall, the median TAT was 17 calendar days (range 4–50 calendar days) from test request to final molecular report. During the timeframe of the analysis (25 months), the number of test demands had increased, in association with a decrease in TAT. In the first trimester, 12 requests had been handled with a median TAT of 33 calendar days, in the last trimester, 46 requests had been processed with a median TAT of 14 calendar days.

### 2.2. Tumor BRCA1 and BRCA2 Alterations Distribution

**Figure 1:**
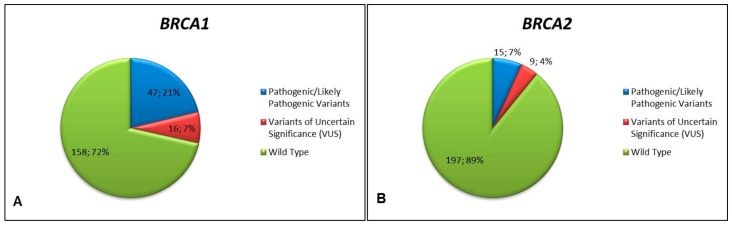
Tumor *BRCA1* (**A**) and *BRCA2* (**B**) status in the study cohort according to variant classification. Pie charts representing the distribution (number of cases; percentage) of *BRCA1* (**A**) and *BRCA2* (**B**) variants among the 221 successfully analyzed. Variant classification is color-coded according to the legend on the right.

**Figure 2:**
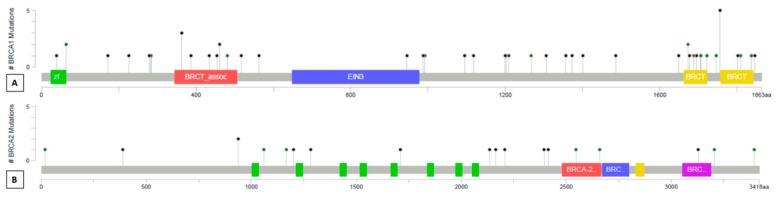
Lollipop plots of mutation distribution on BRCA1 (**A**) and BRCA2 (**B**) protein and domains. The diagrams linearly represent BRCA1/2 protein domains (*x*-axis). BRCA1 domains: green, C3HC4-type RING finger; red, serine-rich domain associated with BRCT; blue, ethylene insensitive 3; yellow, BRCA1 C terminus domain. BRCA2 domains: green, BRCA2 repeats; red, BRCA2 helical; blue, BRCA2 oligonucleotide/oligosaccharide-binding domain 1; yellow, tower; purple, BRCA2 oligonucleotide/oligosaccharide-binding domain 3. Each mutation is represented by single lollipop; the stick lengths indicate mutation frequency (*y*-axis), and dots are color-coded according to alteration type: green, missense mutations; black, truncating mutations (frameshift or nonsense mutations); brown dots, in-frame mutations. Intronic variants are not reported. Graphs created using Mutation Mapper tool, cBioportal (http://www.cbioportal.org/mutation_mapper) and manually curated. BRCA1: RefSeq: NM_007294, Ensembl: ENST00000357654, CCDS: CCDS11453, UniProt: BRCA1_HUMAN. BRCA2: RefSeq: NM_000059, Ensembl: ENST00000380152, CCDS: CCDS9344, UniProt: BRCA2_HUMAN.

Among the 221 cases successfully analyzed, 62 (28.1%) cases harbored tumor *BRCA1* or *BRCA2* pathogenic/likely pathogenic mutations. Twenty-five (11.3%) cases harbored a variant of uncertain significance (VUS), including three cases with *BRCA2* VUS and a concurrent *BRCA1* pathogenic mutation. No other co-occurrence of pathogenic or likely pathogenic mutations was observed. Overall, 87 variants were identified (*BRCA1 n* = 63, 72.4%; *BRCA2 n* = 24, 27.6%), with a median coverage of 1987 reads and a median variant allele frequency of 70%. In detail, 47 pathogenic/likely pathogenic mutations and 16 VUS were identified in *BRCA1*; 15 pathogenic/likely pathogenic mutations and nine VUS occurred in *BRCA2* (Figure 1). The mutations distribution spanned the whole coding sequence of *BRCA1* and *BRCA2* gene, without hotspot regions (Figure 2). Eleven and five alterations affecting the intronic regions were identified in *BRCA1* and *BRCA2*, respectively.

### 2.3. Tumor BRCA1/2 Status According to Clinicopathological Characteristics

The *BRCA1* and *BRCA2* mutations prevalence according to clinicopathological parameters was reported in Table 1. The majority of *BRCA1/2* alterations were identified in primary tumors, whereas only two clinically significant mutations were identified in the sample from the recurrence site. In the high-grade ovarian serous carcinoma subgroup, 31.4% of cases harbored a clinically significant mutation (*n* = 61 cases, 47 *BRCA1* and *n* = 14 *BRCA2*), whereas only one likely pathogenic *BRCA2* alteration was detected in non-high-grade serous carcinoma. Actually, the histotype was the only clinicopathological parameter showing statistically significant association with the presence of *BRCA1/2* mutation (*p*-value = 0.005) (Table 2). However, a higher frequency of *BRCA1/2* mutations was identified among patients with the tumor BRCA test requested at least 6 months after diagnosis without disease-relapse (34.3%) as compared with patients with the test performed at diagnosis or disease relapse (29.3% and 16.2%, respectively). Eleven women with a positive family history previously underwent surgery for breast cancers, and in five cases *BRCA1* (*n* = 4) or *BRCA2* (*n* = 1), pathogenic alterations were detected in the ovary tumor samples.

### 2.4. Tumor and Germline BRCA Tests Concordance

Germline *BRCA1/2* status was available for 62 patients, including 28 cases with tumor BRCA status positive (23 pathogenic/likely pathogenic alterations and five VUS). The tumor and germline BRCA test results are reported in Table 3. Overall, the concordance between tumor and germline BRCA tests was 87.1% (54 of 62), and the negative predictive value of the tumor test was 100%, as all the negative tumor cases had a negative germline test result. All the discordant cases were re-tested in an independent analysis: the specimens were re-evaluated and if available, a different sample was used for the re-test. In all the cases, the tumor BRCA results were confirmed. Five pathogenic/likely pathogenic alterations (*BRCA1 n* = 4 and *BRCA2 n* = 1) and three VUS in *BRCA1* were detected in the tumor specimens only and were considered somatic mutations.

### 2.5. Tumor BRCA Test Workflow in Clinical Diagnostic Setting

**Figure 3:**
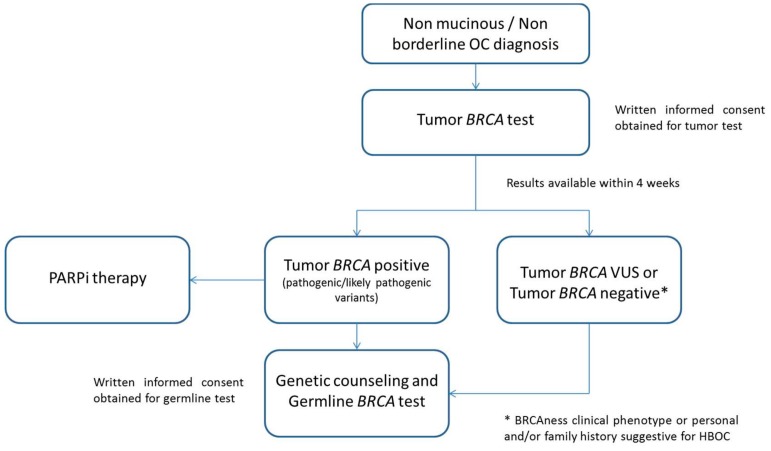
Tumor BRCA test workflow. OC: ovarian cancer; PARPi: poly (adenosine diphosphate (ADP)-ribose) polymerase inhibitors; HBOC: hereditary breast and ovarian cancer.

In the present study, we proposed a workflow for the analysis of tumor *BRCA1/2* status in all patients with a diagnosis of nonmucinous and nonborderline EOC, according to the recently updated AIOM (Associazione Italiana di Oncologia Medica)/SIGU (Società Italiana di Genetica Umana) /SIBIOC (Società Italiana di Biochimica Clinica e Biologia Molecolare Clinica )/SIAPEC-IAP (Patologica e Citologia Diagnostica–italian division of International Academy of Pathology) guidelines [13] (Figure 3). Once the pathological report confirms the diagnosis, the gynecologic oncologist requests the tumor BRCA test upon discussion with the patients and written information on the potential test results and implications. The specific informed consent is collected by the clinician and forwarded with the BRCA test request to the Molecular Diagnostic Unit, where the tumor BRCA test is performed. The result is validated by the molecular biologist and pathologist in a timeframe expected of 4 weeks or less. The patient receives and discusses the test result with the gynecologic oncologist. Patients harboring pathogenic/likely pathogenic tumor *BRCA1/2* mutations may be eligible for PARPi therapy, according to clinical characteristics, and directed to genetic counseling, during which a peripheral blood sample will be tested to evaluate the germline nature of the alteration identified. In the presence of a germline variant, prevention measures and the extension of the genetic counseling to the family members is proposed, according to the institutional procedures. Genetic counseling is advised even for patients harboring variants of unknown significance in the tumor or with a negative tumor test result if they have a suggestive clinical phenotype such as a BRCAness clinical phenotype [14] or their family history is suspicious for hereditary breast and ovarian cancer (HBOC) syndrome. For these patients, the geneticist evaluates the appropriateness of germline BRCA testing as sequencing of *BRCA1/BRCA2*, including the evaluation of large rearrangements or the complementary test seeking for large rearrangements only.

## 3. Discussion

To date in Italy, the PARPi olaparib is approved by Agenzia Italiana del Farmaco (AIFA) as maintenance treatment after a complete or partial response to platinum-based chemotherapy for patients with recurrent high-grade serous ovarian carcinoma, carcinoma of the fallopian tube, or primary peritoneal carcinoma, with germline or somatic *BRCA1/2* mutation [15]. In addition, the results of SOLO1 trial in women with newly diagnosed advanced EOC and a *BRCA1/2* mutation may lead to BRCA testing at the time of diagnosis [7]. Hence, the introduction of a diagnostic test able to identify both germline and somatic *BRCA1/2* alterations in tumor specimens has become necessary to guide treatment choice, even right after the surgery. In this study, we showed the clinical feasibility of an NGS-based tumor BRCA test and we proposed a BRCA test workflow to improve the clinical management of women with EOC.

There are critical pre-analytical and analytical issues in the clinical implementation of an NGS-based BRCA tumor test that are related to (i) the nature of FFPE archival specimens and (ii) the interpretation of NGS results, including mutation identification and classification [8,9,16]. Previous studies have shown that next-generation sequencing analyses represent robust and efficient methods for the detection of *BRCA1/2* alterations from FFPE tumor tissue, including ovarian cancer specimen [10,11,12]. In particular, two studies validated the use of the NGS panel “Oncomine BRCA Research Assay” for tumor BRCA testing, performed on FFPE samples from ovarian cancers [12,17]. Here, we demonstrated the feasibility of the implementation of the NGS-based BRCA tumor tests in a clinical molecular diagnostic setting. All the FFPE samples met the criteria for NGS analysis, with a BRCA tumor test success rate of 99.1%. The technology was robust and fully automatized. Given that at least eight samples have to be analyzed at the same time, with increasing requests of the tumor BRCA test, the TAT from request to results has been reduced to a median of 17 calendar days. This timeframe was suitable for clinical purposes, considering both relapse treatment and maintenance therapy in newly diagnosed cancers.

In our study, 28.1% and 11.3% of cases harbored *BRCA1/2* pathogenic/likely pathogenic alterations and VUS, respectively. The mutations distribution in *BRCA1* and *BRCA2* genes was similar to the data obtained from larger cohorts [18,19]. No hotspot mutations have been identified, as expected in a study population originating from all over Italy. We confirmed the association between the *BRCA1/2* positive status and high-grade serous carcinoma histological subtype. The frequency of *BRCA1/2* mutations was higher in patients with the tumor test requested at least 6 months after diagnosis without disease-relapse. Although not statistically significant, this difference may be related to the favorable prognosis and platinum-sensitivity of tumor with *BRCA1/2* mutations [18]. No other clinical correlation could be evaluated due to the small number of the cases with *BRCA1/2* mutation and an appropriate follow-up.

Compared with germline *BRCA1/2* variant testing, the analysis on tumor tissue allow to identify also somatic alterations. These mutations account for a not insignificant proportion of *BRCA1/2* mutated EOC (3.5–39%), depending on patient selection and histological subtype [18,19,20,21,22]. In the study cohort, considering the subgroup of patients with an available germline test, we detected a quota of potential somatic alterations occurring during the tumor development. In detail, 5/23 (21.7%) pathogenic/likely pathogenic mutations were identified in tumor only. These alterations would not be pinpointed if only a germline blood-based BRCA test was performed. Moreover, as recently reported by Janice Kwon, tumor BRCA testing may represent a cost-effective method of triaging women with EOC for genetic counseling and a confirmatory germline test to identify *BRCA1/2* mutations carriers [23].

We proposed a BRCA test workflow for women with EOC that involved pathologists, biologists, oncologists, surgeons, and geneticists. The aim was first to offer to the patients personalized therapies both in relapse setting and in first-line maintenance treatment. Secondarily, a primary tumor BRCA screening would hasten the germline test and so accelerate the genetic counseling process, for specific cases. Indeed, for patients with BRCA-positive tumor, the germline tests could be at first instance an assessment in the peripheral blood of the alterations identified in the tumor. Thus, the germline or somatic nature of the alteration would be ascertained with different burdens, in terms of genetic counseling. In this workflow, every patient with a diagnosis of nonmucinous and nonborderline EOC would be tested. This may allow the identification of patients with a *BRCA1/2* germline variant even in the absence of a family history suspicious for HBOC, that otherwise would never be included in germline BRCA screening. In the systemic review, Arts-de Jong et al. reported a 6.2% mean probability of finding a germline *BRCA1/2* mutation in a patient with ovarian cancer and without a positive family history for breast and/or ovarian cancer [24]. In the present study, 23 patients with a negative family history had a BRCA tumor test positive for pathogenic/likely pathogenic alteration. Among these, four were confirmed as germline variant by a peripheral blood sample test.

The workflow we described could be a “place to start” for clinical management of women with EOC. Nevertheless, even if the negative predictive value of the tumor BRCA test for identifying germline variants was 100%, it is undisputed that the clinicians should individually evaluate each patient. First, the potential limitations of the tumor test should be considered, including the issue of accurate identification of large rearrangements as well as the tumor heterogeneity associated with the neoplastic growth [25,26]. Indeed, in our series, one case showed a *BRCA1/2* negative status in the primary tumor and a subsequent *BRCA1/2* positive relapse. Second, different studies reported on the phenomenon of “revertant phenotype”. Although it was not observed in our series, this phenomenon cannot be excluded a priori [27,28,29]. Third, we could analyze the concordance between tumor and germline BRCA tests only in a small subgroup of patients (*n* = 62/221). For these reasons, we cautiously suggest performing genetic counseling even when the tumor test is negative but the family history is suggestive for BRCAness phenotype or the patients had a personal and/or family history suggestive for HBOC syndrome.

The management of uncertain results of tumor tests remains controversial. Specific guidelines are needed for the correct handling of these results by both the gynecologic oncologist for therapeutic purposes and the clinical geneticist for genetic risk assessment. In our view, patients should be informed about the need for updates regarding the possibility of reclassification of variants of unknown significance over time.

This study has potential limitations. First, the NGS BRCA panel is currently unsuitable for a reliable measurement of copy number variants in FFPE specimens (large rearrangements accounting for less than 10% of *BRCA1/2* alterations). At the present time, this pitfall is linked to the PCR enrichment methods and bioinformatics algorithms that may overcome this limit are expected. Second, a higher rate of base call error, especially in the homopolymeric region has been reported with the amplicon-based and semiconductor NGS technology as compared with other platforms [30,31,32]. However, in their validation cohort of 43 samples previously analyzed with Illumina platform and/or by Sanger sequencing, Shin et al. did not identify any false-positive variant calls in the homopolymer region, considering an allele frequency of ≥20% [17]. The authors concluded that the new Ion Torrent sequencer with an optimize bioinformatics pipeline may represent an accurate platform for the analysis of *BRCA1/2* genes [17]. Furthermore, to improve the quality of variant call and reduce the error rate, we performed every sequencing run in duplicate on different chips. Third, given the short follow-up time and the small number of patients with *BRCA1/2* mutations, we were not able to evaluate any correlation between *BRCA1/2* status and clinical outcomes. Further studies with larger cohorts and adequate follow-up are required to assess the clinical impact of our BRCA workflow. Finally, we have not investigate the presence of alterations in other genes of the homologous recombination system. Although this evaluation may have an important clinical impact, we focused on *BRCA1/BRCA2* analysis according to the current standard of care requested for predictive purpose.

## 4. Materials and Methods

### 4.1. Study Population

The study population included 223 patients with EOC referred to the Molecular Diagnostics Unit of the European Institute of Oncology from October 2016 to October 2018 for tumor *BRCA1/2* analysis. The tumor BRCA test was required by gynecologic oncologists after the diagnosis of nonmucinous and nonborderline EOC, according to the test guidelines [13]. Each patient gave written informed consent to the tumor BRCA test, form institutional approved and encoded as GNM.MO.5936.B, revised version 23.10.2017.

The clinicopathological characteristics of the study cohort are summarized in Table 4. The clinical data were collected from medical records. The histological subtype and TNM staging were determined by gynecologic pathologists according to the current World Health Organization (WHO) classification of tumors of the female reproductive organs and the 8th Edition American Joint Committee on Cancer (AJCC) Staging Manual [33,34]. The family history was considered positive when at least one first degree relative had a *BRCA1*- or *BRCA2*-related cancer diagnosis. The test request was submitted immediately after the histopathological diagnosis for 123 patients, after 6 months or more from diagnosis for 35 patients without disease relapse, and at the relapse for 37 patients. For 28 patients, these data were not available. For a subgroup of patients (*n* = 62), germline *BRCA1/2* status was also available, as previously assessed with peripheral blood testing.

### 4.2. Next-Generation Sequencing Analysis of BRCA Status in Tumor Samples

DNA was extracted from representative formalin-fixed paraffin-embedded (FFPE) tumor tissues block as previously reported [35]. One hundred and ninety-two (86.1%) cases had FFPE tissue blocks internally stored, whereas for 31 (13.9%) cases, FFPE material was obtained from external institutions. In 134 cases (60.1%), manual macrodissection with a sterile scalpel was performed before nucleic acid isolation. The median tumor cell content was 60% (range 10–90%). *BRCA1/2* status was evaluated using the next-generation sequencing (NGS) panel “Oncomine BRCA Research Assay” (ThermoFisher, Waltham, MA, USA), following the manufacturer’s instructions. Briefly, 10 ng of genomic DNA was used for the library preparation, and the subsequent chip loading performed automatically on the Ion Chef System (ThermoFisher). The sequencing run was done in duplicate using Ion S5 System (ThermoFisher). Data were analyzed using the Ion Reporter Analysis Software. Only mutations with an allele frequency ≥5% and with adequate quality metrics were reported [36]. Each clinically relevant alteration was visually inspected using the Integrative Genomics Viewer (IGV) software (Broad Institute and the Regents of the University of California, Cambridge, MA, USA). No specific algorithm for the detection of large insertion/deletion was available for FFPE tissue analysis, therefore, no copy number variant (CNV) was reported. The mutations were classified in a five-class system according to the IARC (International Agency for Research on Cancer) clinical classification and depending on the ENIGMA consortium revision [37]. The BRCA Exchange database was consulted for variant classification [38]. If the variant clinical significance was reported as “not yet reviewed” by ENIGMA consortium, other public databases were questioned, such as ClinVar [39] or Leiden Open source Variation Database (LOVD) [40]. The analytical workflow of this tumor BRCA test was previously validated on retrospective cases with known *BRCA1/2* status under an inter-laboratory validation program.

### 4.3. Statistical Analysis

Statistical analysis was carried out using SPSS Statistic 25 software (IBM, New York, NY, USA). Chi-Square test with Yates correction was used for data comparison of categorical variables. *p*-values < 0.05 were considered statistically significant.

## 5. Conclusions

In conclusion, we showed that the NGS-based tumor BRCA test may be used in the molecular diagnostic setting for all patients with nonmucinous and nonborderline EOC. The test is suitable for the analysis of FFPE specimens and generated reliable results in a timeframe compatible with clinical needs. We proposed a “BRCA test workflow” that we followed for an integrated clinical and diagnostic management of patients with EOC, in which the tumor BRCA test may act as a compelling tool to drive a personalized treatment of these women.

## Figures and Tables

**Table 1:** Tumor *BRCA1* and *BRCA2* alterations distribution according to the clinicopathological parameters of the 221 cases successfully analyzed.

Clinicopathological Features	*BRCA1*			*BRCA2*		
	Pathogenic/Likely Pathogenic Mutations	VUS	WT	Pathogenic/Likely Pathogenic Mutations	VUS	WT
**Tumor Site**						
Primary tumors (*n* = 200)	46 (23.0%)	13 (6.5%)	141 (70.5%)	14 (7.0%)	7 (3.5%)	179 (89.5%)
Metastases/Recurrences (*n* = 21)	1 (4.8%)	3 (14.3%)	17 (81.0%)	1 (4.8%)	2 (9.5%)	18 (85.7%)
**Histologycal Subtype**						
High-grade serous carcinoma (*n* = 194)	47 (24.2%)	16 (8.2%)	131 (67.5%)	14 (7.2%)	7 (3.6%)	173 (89.2%)
Non-serous carcinoma/subtype not defined (*n* = 27)	0	0	27 (100%)	1 (3.7%)	2 (7.4%)	24 (88.9%)
**Pathological Staging**						
T1 (*n* = 15)	2 (13.3%)	0	13 (86.7%)	2 (13.3%)	2 (13.3%)	11 (73.3%)
T2 (*n* = 29)	6 (20.7%)	1 (3.4%)	22 (75.9%)	3 (10.3%)	0	26 (89.7%)
T3 (*n* = 116)	25 (21.6%)	8 (6.9%)	83 (71.6%)	4 (3.4%)	3 (2.6%)	109 (94.0%)
NA (*n* = 61)	14 (23.0%)	7 (11.5%)	40 (65.6%)	6 (9.8%)	4 (6.6%)	51 (83.6%)
N0 (*n* = 39)	5 (12.8%)	0	34 (87.2%)	3 (7.7%)	4 (10.3%)	32 (82.1%)
N1 (*n* = 65)	18 (2.7%)	6 (9.2%)	41 (63.1%)	2 (3.1%)	1 (1.5%)	62 (95.4%)
NX (*n* = 56)	10 (17.9%)	3 (5.4%)	43 (76.8%)	4 (7.1%)	0	52 (92.9%)
NA (*n* = 61)	14 (23.0%)	7 (11.5%)	40 (65.6%)	6 (9.8%)	4 (6.6%)	51 (83.6%)
**Family History**						
Positive (*n* = 93)	24 (25.8%)	8 (8.6%)	61 (65.6%)	7 (7.5%)	6 (6.5%)	80 (86.0%)
Negative (*n* = 101)	16 (15.8%)	7 (6.9%)	78 (77.2%)	7 (6.9%)	2 (2.0%)	92 (91.1%)
NA (*n* = 27)	7 (25.9%)	1 (3.7%)	19 (70.4%)	1 (3.7%)	1 (3.7%)	25 (92.6%)
**Time of Test Request**						
At pathological diagnosis (*n* = 123)	28 (22.8%)	8 (6.5%)	87 (70.7%)	8 (6.5%)	5 (4.1%)	110 (89.4%)
At least 6 months after pathological diagnosis without relapse (*n* = 35)	9 (25.7%)	2 (5.7%)	24 (68.6%)	3 (8.6%)	3 (8.6%)	29 (82.9%)
At relapse (*n* = 37)	4 (10.8%)	5 (13.5%)	28 (75.7%)	2 (5.4%)	1 (2.7%)	34 (91.9%)
NA (*n* = 26)	6 (23.1%)	1 (3.8%)	19 (73.1%)	2 (7.7%)	0	24 (92.3%)

The bold terms referred to the clinicopathological feature investigated.

**Table 2:** Correlation between BRCA status and clinicopathological features.

Clinicopathological Features	BRCA MUTATED (Phatogenic/Likely Pathogenic Mutations)	BRCA WT *	*p* Value **
**Tumor Site**			
Primary tumors (*n* = 200)	62 (31.0%)	138 (69.0%)	0.07
Metastases/Recurrences (*n* = 21)	2 (9.5%)	19 (90.5%)	
**Histologycal Subtype**			
High-grade serous carcinoma (*n* = 194)	61 (31.4%)	133 (68.6%)	0.005
Non-serous carcinoma/subtype not defined (*n* = 27)	1 (3.7%)	26 (96.3%)	
**Pathological Staging**			
T1 (*n* = 15)	4 (26.7%)	11 (73.3%)	0.716
T2 (*n* = 29)	9 (31.0%)	20 (69.0%)	
T3 (*n* = 116)	29 (25.0%)	87 (75.0%)	
NA (*n* = 61)	20 (32.8%)	41 (67.2%)	
N0 (*n* = 39)	8 (20.5%)	31 (79.5%)	0.518
N1 (*n* = 65)	20 (30.8%)	45 (69.2%)	
NX (*n* = 56)	14 (25.0%)	42 (75.0%)	
NA (*n* = 61)	20 (32.8%)	41 (67.2%)	
**Family History**			
Positive (*n* = 93)	31 (33.3%)	62 (66.7%)	0.258
Negative (*n* = 101)	23 (22.8%)	78 (77.2%)	
NA (*n* = 27)	8 (29.6%)	19 (70.4%)	
**Time of Test Request**			
At pathological diagnosis (*n* = 123)	36 (29.3%)	87 (70.7%)	0.33
At least 6 months after pathological diagnosis without relapse (*n* = 35)	12 (34.3%)	23 (65.7%)	
At relapse (*n* = 37)	6 (16.2%)	31 (83.8%)	
NA (*n* = 26)	8 (30.8%)	18 (69.2%)	

* Wild Type (WT) or variants of uncertain significance (VUS) variants. ** *p*-value < 0.05, statistically significant Chi-Squared Test. The bold terms referred to the clinicopathological feature investigated.

**Table 3:** Correlations between tumor and germline *BRCA1/2* status (*n* = 62).

Tumor BRCA Test		Germline BRCA Test		
		Positive		Negative
		Pathogenic/Likely Pathogenic	VUS	
**Positive**	Pathogenic/Likely Pathogenic	18 (78.3%)	/	5 (21.7%)
	VUS	/	2 (40%)	3 (60%)
**Negative**		/	/	34 (100%)

**Table 4:** Clinicopathological characteristics of the study population.

Clinicopathological Features	*n* (%)
**Time of Test Request**	
At pathological diagnosis	123 (55.2%)
At least 6 months after pathological diagnosis without relapse	35 (15.7%)
At relapse	37 (16.6%)
NA	28 (12.6%)
**Histological Subtype**	
High-grade serous carcinoma	195 (87.4%)
Endometroid carcinoma	16 (7.2%)
Clear cell carcinoma	9 (4%)
Carcinoma—subtype not defined	3 (1.3%)
**TN Pathological Staging**	
T1	15 (6.7%)
T2	29 (13%)
T3	116 (52%)
NA	63 (28.3%)
N0	39 (17.5%)
N1	65 (29.1%)
NX	56 (25.1%)
NA	63 (28.3%)
**Specimen Analyzed**	
Primary Tumor	201 (90.1%)
Ovary localization	169 (75.8%)
Extra-ovary localization	32 (14.3%)
Pelvic localization	14 (43.8%)
Extra-Pelvic localization	12 (37.5%)
NA	6 (18.7%)
Metastasis/recurrence	22 (9.9%)
Lymph nodal metastasis	3 (13.6%)
Extra lymph nodal metastasis	19 (86.4%)
**Neoadjuvant Treatment**	
Yes	46 (20.6%)
No	114 (51.1%)
NA	63 (28.3%)
**Surgery**	
R0 - no residual disease	135 (60.5%)
R+ < 1 cm	42 (18.8%)
R+ > 1 cm	19 (8.5%)
NA	27 (12.1%)
**Family History**	
Positive	93 (41.7%)
Negative	101 (45.3%)
NA	29 (13%)

The bold terms referred to the clinicopathological features investigated.
